# Effects of Strawberry Tree Water Leaf Extract and Arbutin on Biochemical Markers and DNA Integrity in Brain Cells of Lewis Rats

**DOI:** 10.3390/toxics12080595

**Published:** 2024-08-16

**Authors:** Vesna Benković, Dora Vuković, Iva Đelatić, Vanja Popović, Karlo Jurica, Fabijan Knežević, Irena Brčić Karačonji, Ana Lucić Vrdoljak, Nevenka Kopjar

**Affiliations:** 1 Faculty of Science, University of Zagreb, 10000 Zagreb, Croatia; 2 Special Security Operations Directorate, Ministry of the Interior, 10000 Zagreb, Croatia; juricakarlo@gmail.com; 3 School of Medicine, Catholic University of Croatia, 10000 Zagreb, Croatia; knezevicfabijan@yahoo.com; 4 Institute for Medical Research and Occupational Health, 10000 Zagreb, Croatia; ibrcic@imi.hr (I.B.K.); alucic@imi.hr (A.L.V.); nkopjar@imi.hr (N.K.); 5 Faculty of Health Studies, University of Rijeka, 51000 Rijeka, Croatia

**Keywords:** *Arbutus unedo* L., arbutin, leaf extract, lipid peroxidation, antioxidant enzymes, alkaline comet assay, rat brain cells

## Abstract

There is growing evidence that arbutin and plant extracts rich in arbutin, such as extracts of the strawberry tree (*Arbutus unedo* L.), exert a range of beneficial effects, including cyto- and genoprotective properties. This study evaluated the effects of strawberry tree water leaf extract (STE) and arbutin in the brain tissue of Lewis rats. STE or arbutin were administered per os to male and female rats at a dose of 200 mg/kg body weight/day for 14 or 28 days. Treatment outcomes were evaluated using biochemical markers (lipid peroxidation and the activities of the antioxidative enzymes catalase and superoxide dismutase). The effects of the tested substances on DNA integrity in brain cells were evaluated using the alkaline comet assay. The results suggest a high biocompatibility of both tested substances with rat brain tissue. No significant harmful disturbances were observed in the oxidative/antioxidative status or impairments of DNA integrity in the rat brain cells. Nearly all post-treatment values were within tolerable limits as compared to the matched control rats. Such encouraging findings support further research using other subtle biomarkers to clarify the safety aspects of arbutin and STE prior to the development of specific nutraceutical products.

## 1. Introduction

There is a widely accepted paradigm that many plants and herbal extracts have protective and prophylactic potential in various pathological conditions owing to high contents of specific bioactive ingredients. In recent years, there has been a growing body of evidence concerning the beneficial health effects of arbutin (4-hydroxyphenyl-*β*-D-glucopyranoside), a key component in herbal products that primarily originates from bearberry [*Arctostaphylos uva-ursi* (L.) Spreng.] and strawberry tree (*Arbutus unedo* L.). In addition to its traditional urinary antiseptic role [1,2,3], arbutin has proven to be a highly promising compound in studies focused on radiation protection [4]. Furthermore, it has shown beneficial effects on the skin as a depigmentation agent [5], and recent studies have also highlighted its neuroprotective properties and counteractive effects against oxidative stress [6,7,8,9,10,11,12]. To date, our research group has examined the effects of arbutin and strawberry tree water leaf extract (STE) rich in arbutin on different experimental models under in vitro [13] and in vivo conditions [14,15,16]. Findings of the in vitro study showed the high biocompatibility of STE and arbutin with human peripheral blood lymphocytes [13], while in vivo studies focused on a rat model showed their favourable effects on haematological parameters [14], without the induction of hepatotoxic [15] or nephrotoxic effects [16]. In our previous in vitro research, we found that STE had concentration-dependent antioxidant potential. However, we have not investigated the relationship between oxidative stress and arbutin or STE in an in vivo model so far.

Preparations based on strawberry tree leaves have the potential to complement or even substitute traditionally used bearberry leaves. However, unlike bearberry, whose beneficial effects have been proven and reported in many studies to date, the toxicological and safety profile of strawberry tree still requires detailed study on various experimental systems.

We hypothesised that, due to the high biocompatibility of arbutin and STE previously observed at the level of single cells and tissues [13,14,15,16], similar non-harmful effects could be expected in rat brain tissue. To test this hypothesis, we investigated the outcomes of 14- vs. 28-day exposure of Lewis rats of both sexes to arbutin or STE administered at a daily dose of 200 mg/kg body weight (b.w.). The methodology included measuring the levels of the basic biochemical parameters related to oxidative stress [lipid peroxidation and the activity of the antioxidative enzymes catalase (CAT) and superoxide dismutase (SOD)] in the brain tissue of exposed animals coupled with a study of DNA damage evaluated in single cells using the alkaline comet assay.

The results obtained provide new insight into the levels of biochemical and molecular markers that sustain beneficial effects of the tested compounds on rat brain tissue. An additional aim of this study was to contribute to the knowledge of neuroprotective effects worthy of future research and potential applications of arbutin-rich products as nutritional/dietary supplements.

## 2. Materials and Methods

### 2.1. Materials

The chemicals and reagents used in this study were purchased from Sigma-Aldrich (Steinheim, Germany) unless otherwise stated.

*Arbutus unedo* L. plant material was collected on the island of Mali Lošinj (Croatia). More details and procedures involved in the extract preparation were reported by Jurica et al. [13]. Briefly, strawberry tree leaves were air-dried and ground in a laboratory mill. Powdered leaf samples (3 g) were extracted with water (80 mL) in an ultrasonic bath at 50 °C for 60 min. The mixture was filtered through a 0.45 µm filter paper and freeze-dried.

### 2.2. Experimental Design

#### 2.2.1. Ethical Statement

This experiment was part of a large study approved by the Ethics Committee of the Faculty of Science, University of Zagreb, Croatia (approval number: 251-58-10617-14-104). All protocols in the study were compliant with Directive 2010/63/EU. The study was carried out in compliance with the legal and ethical principles applicable in the Republic of Croatia and with international animal welfare standards.

#### 2.2.2. Laboratory Animals

The selected experimental models were Lewis rats of both sexes (N = 48). The rats were obtained from the breeding colony of the Division of Animal Physiology, Department for Biology, Faculty of Science, University of Zagreb, Croatia. The rats were maintained in a steady-state microenvironment at 22 ± 1 °C, with 50–70% humidity with a 12 h light/dark photoperiod and free access to water and standard GLP-certified food (4RF21; Mucedola, Settimo Milanese, Milan, Italy). The animals were weighed and randomised into 12 experimental groups (N = 4 rats per group) based on sex (M or F), tested substance (STE, arbutin, or negative control), and duration of treatment (14 or 28 days). At the beginning of the experiment, the animal age was 60 ± 5 days, and the initial average body weight was about 200 g.

#### 2.2.3. Treatment Protocol

The daily dose of arbutin used for testing (200 mg/kg b.w.) was selected in accordance with the risk assessment reports on arbutin [17,18,19], bearing in mind possible metabolic differences between rats and humans. Rats in general tolerate high arbutin doses, i.e., for acute oral exposure, the LD_50_ is 8715 mg/kg b.w. [19]. In contrast, the recommended daily dose of arbutin for adult humans, when taken via herbal preparations, could be up to 800 mg during a maximum period of two weeks [18]. The daily dose of STE was selected according to the relevant literature [17,18,19] regarding the use of the related medicinal plant bearberry [*Arctostaphylos uva ursi* (L.) Spreng].

The strawberry tree (*Arbutus unedo* L.) water leaf extract (STE) used in the study was of known phytochemical composition. Apart from arbutin, 59 phenolics were detected in the STE, with hyperoside and flavan-3-ols being the predominant compounds. More detail on its characterisation was reported by Brčić Karačonji et al. [20]. The arbutin content in the lyophilisate of STE, as determined by high-performance liquid chromatography (HPLC) with diode array detection (DAD), was 1.07% [21].

To prepare the treatment solutions, arbutin and lyophilised STE were dissolved in double-distilled water. Both solutions were administered to rats through an oral gastric cannula for 14 or 28 days, in accordance with the provisions of 407 OECD (Organization for Economic Cooperation and Development) and US EPA (United States Environmental Protection Agency) recommendations for the duration of short-term toxicological treatment [22]. Negative control groups were kept in the same conditions, and those rats received per os an equal volume of double-distilled water.

The detailed experimental schedule is outlined in Table 1.

Animal behaviour, survival, and clinical signs of intoxication were monitored on a daily basis. Body weight was measured at the beginning and end of the experiment (on day 14 or 28 after receiving the first dose of the test substance).

Animals were sacrificed by standard procedure: after anaesthesia with an anaesthetic cocktail [Narketan^VR^ (Vetoquinol SA, BP 189 Lure Cedex, France) (active substance: ketamine) (0.8 mL/kg) and Xylapan^VR^ (*Vetoquinol biowet* Sp. Gorzow, Poland) (active substance: xylazine) (0.6 mL/kg)], the rats were decapitated.

From each rat, brain tissue from the frontal lobe was dissected, rinsed with cold phosphate-buffered saline (PBS), and divided into two sections. The tissue samples used for biochemical analyses were immediately frozen at −20 °C until further processing. Small sections of the frontal lobe were immediately processed according to the protocol for the alkaline comet assay.

### 2.3. Biochemical Analyses

#### 2.3.1. Determination of Total Proteins

The total protein concentration in the brain tissue of the Lewis strain rats was determined using the Lowry protein assay [23]. This method allows for the calculation of the protein concentration in a sample based on a colour change following a specific reaction between tryptophan and tyrosine residues with Folin–Ciocalteu reagent, measured by absorbance at 660 nm. The standard used was bovine serum albumin (BSA) in different concentrations (1.5, 1.25, 1, 0.75, 0.5, 0.25, 0.125, and 0 mg/mL). The obtained values for the protein concentrations in the samples were expressed as mg/mL and were used in further calculations to determine the activity of antioxidant enzymes and the concentration of malondialdehyde (MDA) in brain tissue.

#### 2.3.2. Lipid Peroxidation

The degree of lipid peroxidation was determined by measuring the concentration of MDA in brain cells of the Lewis strain rats. The methodology reported by Jutrić et al. [24] was applied. In this method, the MDA in the sample reacts with thiobarbituric acid (TBA) to generate an MDA-TBA complex. Measurements were performed at a wavelength of 532 nm on a Libra S22 spectrophotometer (Biochrom, UK). The total MDA concentration was calculated from the molar absorption coefficient of the MDA-TBA complex (1.56 × 10^5^ L/mol cm) and expressed as nmol of MDA per mg of protein.

#### 2.3.3. Catalase Activity

Determination of CAT activity in Lewis rat brain tissue was performed using a UV-160 spectrophotometer (Shimadzu, Kyoto, Japan), according to the procedure described by Jutrić et al. [24]. The test is based on monitoring the decrease in absorbance of the reaction mixture during 60 s at 240 nm (UV region) resulting from the enzymatic decomposition of hydrogen peroxide (H_2_O_2_), which shows maximum absorbance at λ = 240 nm. CAT activity was expressed through the extinction coefficient of hydrogen peroxide (ε = 39.4 mM^−1^cm^−1^) as μmol decomposed hydrogen peroxide per minute per milligram protein (μmol H_2_O_2_/min/mg of protein). The results were expressed as units of CAT per milligram protein (U CAT/mg protein).

#### 2.3.4. Superoxide Dismutase Activity

Determination of SOD activity in brain cells of the Lewis strain rats was performed using a UV spectrophotometer UV-160 (Shimadzu), as described by Jutrić et al. [24]. The method is based on the inhibition of cytochrome c reduction in the xanthine/xanthine oxidase system. The measurement was performed at a wavelength of 550 nm. SOD activity was expressed as U/mg protein and was measured as the percentage of inhibition of xanthine oxidase activity.

### 2.4. Comet Assay

The methodological procedures used in this study followed the basic protocol of the alkaline comet assay [25], as described previously, with minor modifications [26,27]. Two replicate slides were prepared per rat and stained with 100 µL ethidium bromide (20 mg/mL) for 10 min. They were observed under 200× magnification using an epifluorescence microscope (Olympus BX50, Olympus, Tokyo, Japan) equipped with appropriate filters. Measurements of comets were performed in individual cells using an image analysis system (Comet Assay IVTM; Instem-Perceptive Instruments Ltd., Suffolk, Halstead, UK). A total of 50 comets per rat were recorded. For comet scoring, random spots were chosen, avoiding the edges. The main descriptors of DNA damage were tail intensity (DNA% in comet tail) and tail length (in micrometres).

### 2.5. Statistical Analysis

Data were analysed using Statistica software, Data Science Workbench, version 14 (TIBCO Software Inc., Palo Alto, CA, USA). For biochemical markers, we first used descriptive statistics, while the comparison between independent samples was performed by Kruskal–Wallis analysis of variance (ANOVA) with Tukey’s test. Numerical data obtained from comet measurements were first processed using descriptive statistics methods. Multiple comparisons between groups were tested using ANOVA on log-transformed data with Tukey’s HSD post hoc test. The level of statistical significance was set at *p* < 0.05.

## 3. Results and Discussion

Overall, the results support the working hypothesis and showed that 14- vs. 28-day exposure of male or female Lewis rats to arbutin or STE (applied at a daily dose of 200 mg/kg b.w.) did not produce detrimental toxic effects in brain tissue. All animals survived the experiments, and there were no signs of systemic toxicity following exposure to the test substances.

A high level of biocompatibility of both tested substances with rat brain tissue was confirmed, with no observance of harmful disturbances to oxidative/antioxidative status or DNA damage in rat brain tissue. These results are comparable with our previous observations regarding the beneficial effects of arbutin and STE on rat livers [15] and kidneys [16].

Detailed insight into the values of biochemical markers and DNA damage descriptors in male and female rats, together with a corresponding discussion, is given below.

### 3.1. Lipid Peroxidation

**Figure 1:**
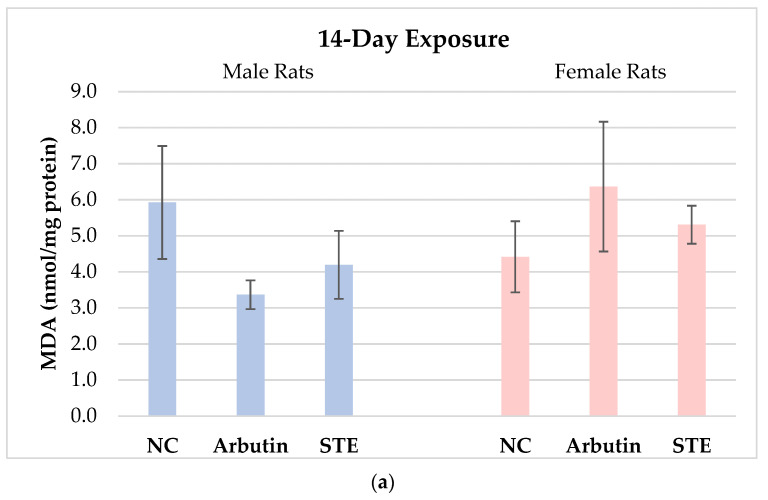

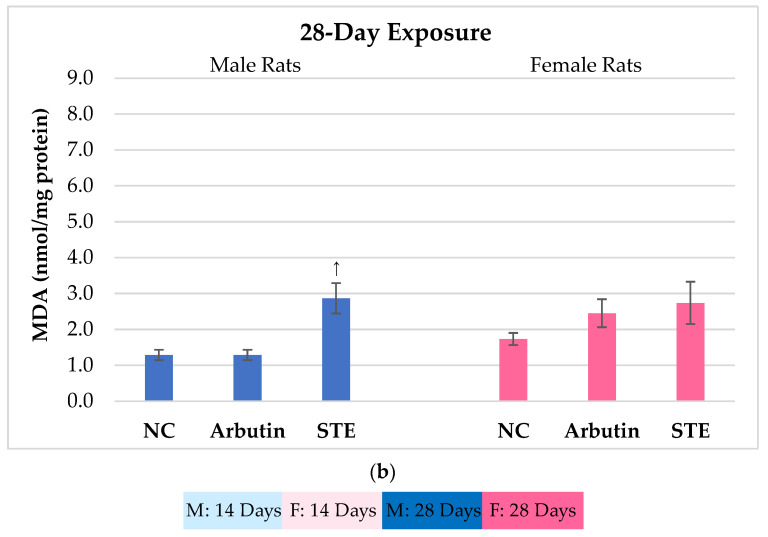
Concentrations of malondialdehyde (MDA) in brain cells of male and female Lewis rats measured after 14-day (**a**) vs. 28-day (**b**) treatment with arbutin or strawberry tree water leaf extract (STE) and in the corresponding negative controls (NC). Values are presented as means ± standard deviations. ↑ Significantly increased (*p* < 0.05) from the matched negative control group after 28-day exposure.

The concentrations of MDA in brain cells of the male and female Lewis rats measured after 14- vs. 28-day treatment with arbutin or STE and in the matched negative controls are reported in Figure 1.

MDA levels in male rats exposed to arbutin or STE for 14 days were insignificantly lower than in the negative controls. In females, MDA values were higher than in the negative controls, though the differences were not statistically significant (Figure 1a).

Extended treatment (28 days) with the tested substances provoked an adaptive response at the cellular level, counteracting oxidative stress, since total MDA levels measured after 28-day exposure in both male and female rats diminished in comparison with the shorter exposure.

However, male rats exposed to STE for 28 days had significantly higher MDA levels compared to the corresponding negative control and arbutin-exposed rats (Figure 1b). It is known that increased levels of MDA indicate overproduction of free radicals in cells. The question arises as to the origin of the free radicals if the rats were given an extract with presumed beneficial properties. The answer may be found within the complex phytochemical profile of the tested extract. Regardless of the overall beneficial effects of many plant extracts, it is clear from the literature that such complex mixtures often contain reactive components that may exhibit pro-oxidative effects under certain circumstances [28,29,30]. The STE used in this study contained 59 phenolics besides arbutin (3050 mg/kg dried leaf weight) [20]. In interpreting the results, we must consider that over the 28-day administration of the extract, complex metabolic reactions unfolded within the rats. Each constituent of the herbal mixture follows its own specific metabolic pathway, which may also involve the generation of reactive intermediates and different types of reactive oxygen species capable of affecting the process of lipid peroxidation. It is well established that arbutin metabolism involves its conversion into the more reactive hydroquinone. As reported [31,32,33], its toxic effects alongside other mechanisms are also mediated by oxidative stress. There are also many reports in the literature regarding the dual nature of phenolic constituents, some of which were present in the STE used here [28,30,34,35].

Still, considering the specific experimental conditions, the observed increase in MDA in the STE-exposed male rats should not be of great concern. Firstly, the duration of exposure in this study was 28 days, which is longer than recommended for the therapeutic use of preparations based on *A. uva-ursi*, which is usually limited to 14 days in continuity. Secondly, the tested daily dose was higher than the recommended therapeutic dose [36]. Finally, a significant increase in MDA was observed only in male rats. It is important to note that the incidence of urinary tract infections in the human male population is much lower than in females [37,38]. It is expected that in real-life scenarios women will be potential consumers of any new preparation against urinary tract infections. Therefore, in terms of possible adverse side effects of the treatment with STE, we stress that no significant side effects were found in the experimental female rats. Nevertheless, the issue of lipid peroxidation in brain cells following exposure to various plant extracts requires further study.

### 3.2. Catalase (CAT) Activity

Catalase plays an important role in protecting cells from oxidative damage, as it mediates the breakdown of hydrogen peroxide into oxygen and water [39,40]. The brain is generally susceptible to oxidative stress due to its high lipid content, high energy needs, and weak antioxidant capacity [41].

Commonly, an increase in CAT activity represents a compensatory or protective reaction to various injuries. Therefore, when testing an extract or single bioactive compound expected to have a beneficial or protective effect, it should not cause significant increases in the activity of this enzyme.

**Figure 2:**
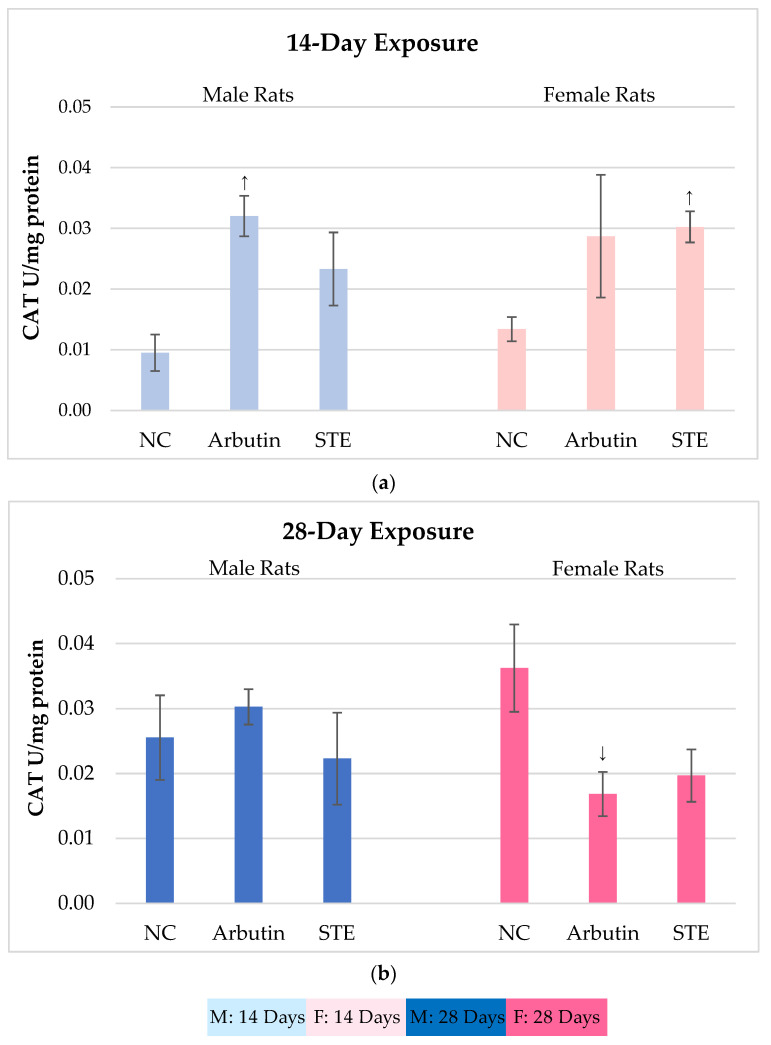
Catalase (CAT) activity in brain cells of male and female Lewis rats measured after 14- vs. 28-day treatment with arbutin or strawberry tree water leaf extract (STE) and in the corresponding negative controls (NC). Values are presented as means ± standard deviations. **↑** Significantly increased (*p* < 0.05) from the matched negative control group after 14-day exposure. ↓ Significantly decreased (*p* < 0.05) from the matched negative control group after 28-day exposure.

Figure 2 shows the results on CAT activity in the brain cells of male and female Lewis rats measured after 14- vs. 28-day treatment with arbutin or STE and in the corresponding negative controls.

After 14-day exposure, significantly increased CAT activity as compared to the controls was observed in the arbutin-treated male rats and the STE-treated female rats (Figure 2a). It is possible that these increases were related to the previously mentioned metabolic conversions of arbutin into hydroquinone and the metabolism of phenolic compounds in STE that may lead to a transient burst in reactive oxygen species (ROS) production.

We found that 28-day exposure to either substance did not produce significant variations in CAT activity as compared to the controls. The exceptions were arbutin-treated females that showed a statistically significant decrease in brain CAT activity compared to the corresponding negative controls (Figure 2b).

Together, the results regarding CAT activity after the treatments with arbutin or STE speak in favour of their biocompatibility at the tested doses. The lower CAT activity measured after both the arbutin and STE treatments over 28 days (i.e., longer than the recommended therapeutic use of preparations based on *A. uva-ursi*) does not suggest induction of oxidative stress, while the extent of potential protective effects requires further study over a broader dosage range.

### 3.3. Superoxide Dismutase (SOD) Activity

Superoxide dismutases (SODs) play a pivotal role in the antioxidant enzyme system that protects cells from ROS. They catalyse the dismutation of deleterious superoxide anion radicals (O_2_^−^) into molecular oxygen and hydrogen peroxide [42,43,44]. Decreased levels of SODs or mutations affecting their catalytic activity may increase the vulnerability of cells to oxidative stress, which is especially deleterious in brain tissue. Thus, any plant extract or single bioactive compound with an expected protective effect should not impair SOD activity in the brain.

**Figure 3:**
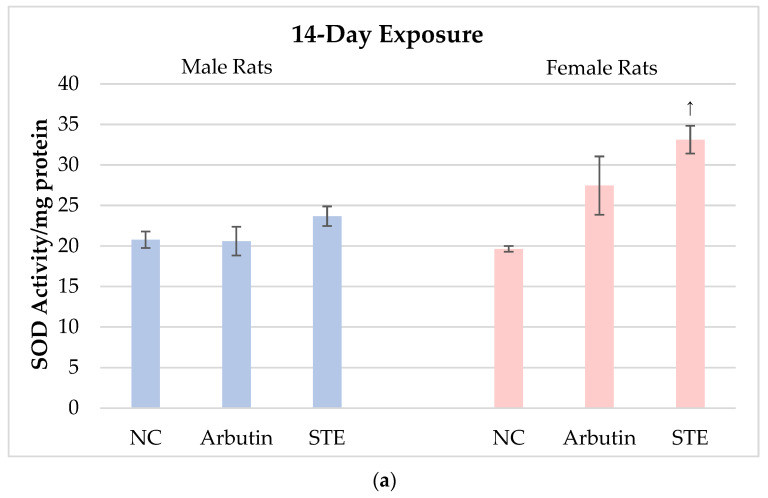

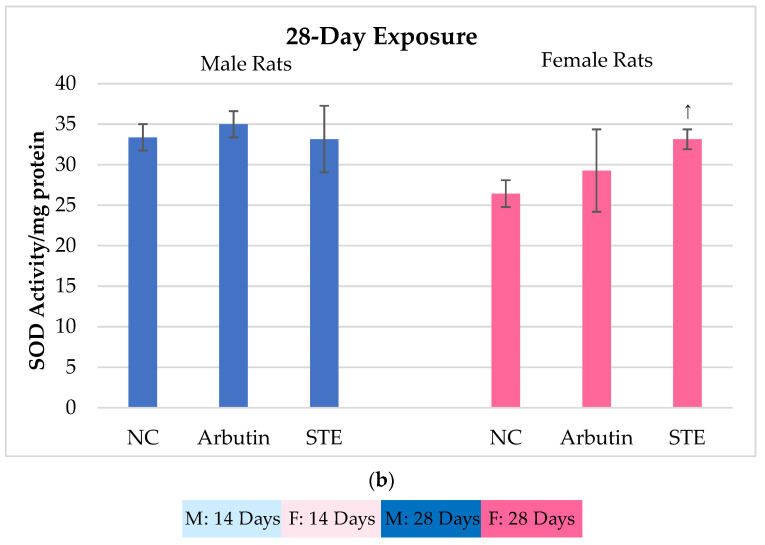
Superoxide dismutase (SOD) activity in brain cells of male and female Lewis rats measured after 14- vs. 28-day treatment with arbutin or strawberry tree water leaf extract (STE) and in the corresponding negative controls (NC). Values are presented as means ± standard deviations. ↑ Significantly increased (*p* < 0.05) from the matched negative control group after 14-day, or 28-day exposure.

Figure 3 shows the results on SOD activity in brain cells of male and female Lewis rats measured after 14- vs. 28-day treatment with arbutin or STE and in the corresponding negative controls.

The results regarding SOD activity after 14- vs. 28-day treatments with arbutin speak in favour of its biocompatibility with brain tissue at the tested doses. These exposures resulted in slight though insignificant variations in SOD activity as compared to the matched controls (Figure 3a,b). STE-treated females showed significant increases in brain SOD activity compared to the corresponding negative controls. It is likely that, in females, repeated exposure to the extract and its complex composition may have stimulated overproduction of ROS, which in turn led to a compensatory reaction of SOD, as seen in its higher activity, though proper interpretation would require further detailed study over a broader dosage range.

### 3.4. Alkaline Comet Assay

Today, there are various tests that can successfully assess the level of DNA instability in cells or nuclei isolated from multiple tissues of rodents exposed to potentially genotoxic agents. Among them, the alkaline comet assay is popular, as it enables sensitive detection of single- and double-strand breaks, alkali-labile sites, alkylated and oxidised bases, DNA-DNA crosslinks, UV-induced cyclobutane pyrimidine dimers, and several chemically induced DNA adducts [45,46,47].

In 2014, the Organisation for Economic Co-operation and Development (OECD) recognised the importance of this test and proposed guidelines for its application in the testing of chemicals (name: Test No. 489: In Vivo Mammalian Alkaline Comet Assay) [48]. Later, the method was further improved and validated, and, recently, detailed protocols were published for its application in a wide variety of cell types, species, and types of DNA damage [47].

The advantage of the comet assay is that it enables a quick and reliable assessment of the occurrence of DNA breaks after treatment. Generally, DNA breaks can originate from direct interactions with DNA or from alkali-labile sites (i.e., apurinic/apyrimidinic sites), or they can be formed during DNA excision repair (as transient DNA strand breaks) [48].

Our study group successfully applied the alkaline comet assay in previous experiments, including experiments focused on the evaluation of DNA damage in liver and kidney cells of Lewis rats exposed to arbutin or STE [15,16]. The results of the latter indicated the low DNA damage potential of both substances at the same doses and treatment durations. Considering that no previous studies have evaluated the levels of DNA damage in brain tissue following exposure to the same substances, the present study provides the first information in that regard.

**Figure 4:**
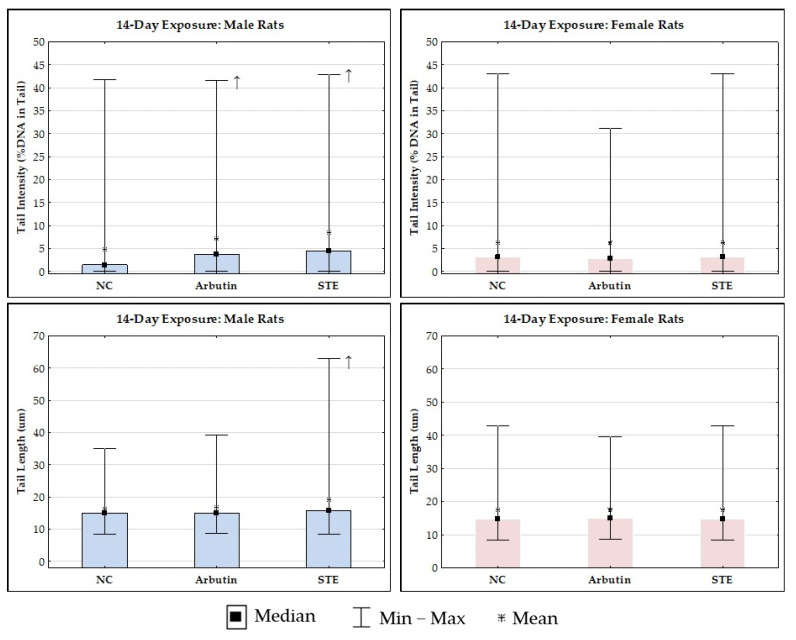
Results of the alkaline comet assay on brain cells of Lewis rats after 14-day treatment with arbutin or strawberry tree water leaf extract (STE), administered per os at a daily dose of 200 mg/kg, and of the respective negative controls. Data are reported as mean values of 200 independent comet measurements per experimental group (50 comets/rat were scored on duplicate slides). Inter-group differences in male or female rats were tested by ANOVA with Tukey’s post hoc HSD test. ↑ Significantly higher values (*p* < 0.05) compared to the negative controls.

**Figure 5:**
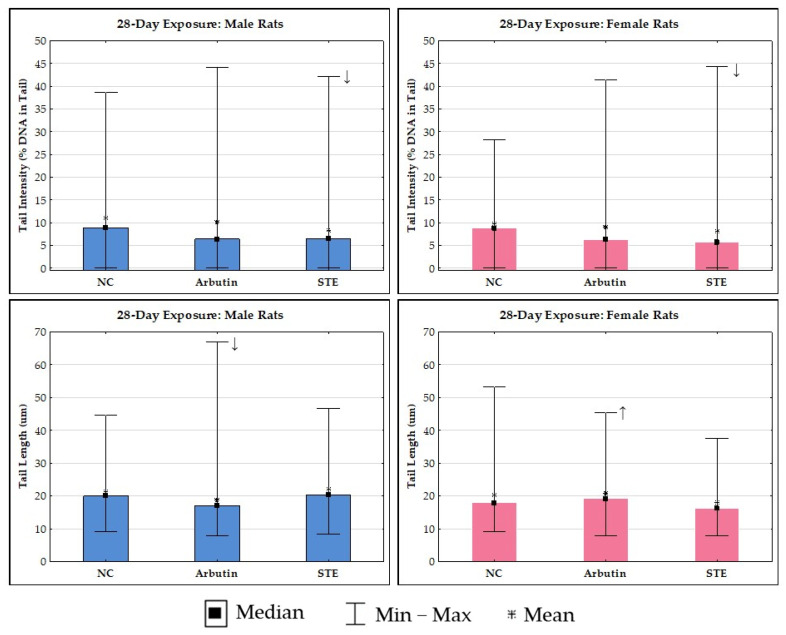
Results of the alkaline comet assay in brain cells of Lewis rats after 28-day treatment with arbutin or strawberry tree water leaf extract (STE), administered per os at a daily dose of 200 mg/kg, and the respective negative controls. Data are reported as mean values of 200 independent comet measurements per experimental group (50 comets/rat were scored on duplicate slides). Inter-group differences between male or female rats were tested by ANOVA with Tukey’s post hoc HSD test. ↓ Significantly lower values compared to the matched negative controls; ↑ significantly higher values (*p* < 0.05) compared to the matched negative controls.

The results of the alkaline comet assay in brain cells are reported in Figure 4 and Figure 5. Control rats of both sexes generally had a low level of spontaneous DNA damage to brain cells.

The outcomes of 14-day exposure at the DNA level of brain cells, along with detailed inter-group comparisons and their significances, are shown in Figure 4.

The treatments did not significantly influence group mean values of tail intensity in the female rats. In the male rats that received the treatment with arbutin, we found a significantly higher value for mean tail intensity compared to the matched negative controls. However, based on our 20+ years of experience using this method, we propose that this difference was more likely due to the very low level of background DNA damage in the negative control rats than to the potentially genotoxic effects of arbutin.

Since group mean values of tail intensity, irrespective of the test substance, were <10% in both male and female rats (which is considered an acceptable level [49]), there is no danger of detrimental genotoxic effects following treatments with arbutin or STE.

The treatments did not significantly influence group mean values of the second descriptor, i.e., tail length in female rats. In male rats after treatment with STE, we found a significantly higher value for mean tail intensity compared to the negative controls. Again, this was likely due to the very low level of background DNA damage in the negative control rats.

The outcomes of 28-day exposure at the DNA level of rat brain cells are shown in Figure 5. The group mean values for tail intensity measured after 28-day exposure for both male and female rats were within the acceptable level range, i.e., <10%. Exposure to arbutin or STE in male and female rats resulted in a reduction in DNA damage compared to the matched negative controls, though this was statistically significant only for the STE-treated groups.

In male rats, 28-day exposure to arbutin resulted in a lower group mean value for tail length compared to the negative control and STE groups. However, quite the opposite trend was observed in female rats. The slightly increased group mean value for tail length following exposure to arbutin can be attributed to the higher sensitivity of DNA in female rat brain cells during prolonged exposure. Continued delivery of arbutin appeared to produce a certain amount of DNA breakage or enhanced repair processes, thereby contributing to the higher values measured with the comet assay.

In general, genomic DNA is prone to various types of DNA damage, starting from single-strand breaks and oxidative damage up to the most harmful double-strand breaks. Fortunately, brain cells possess efficient DNA damage response (DDR) pathways that counteract most damage. Nevertheless, defective DDR can result in toxic genomic rearrangements, transcriptional dysregulation, and the accumulation of unrepaired lesions that finally direct cellular fate to apoptosis, senescence, or uncontrolled cell division [50]. Therefore, the potentially detrimental neurotoxic effects of unknown phytochemical preparations have to be extensively studied. The body of knowledge on the safety of many plant extracts and their beneficial properties for the nervous system is constantly growing, and the neuroprotective effects of many of these extracts are well established [51,52,53,54,55]. However, prior to this study, there was no information on STE or arbutin applied at the doses we tested.

The findings of biocompatibility at the DNA level of brain cells are especially important, since a large part of brain tissue consists of neurons that (in contrast to glial cells) are terminally differentiated cells that cannot divide and replace highly damaged cells affected by apoptosis or necrosis. It should be stated that DNA damage detected by the comet assay can have several outcomes, i.e., it can (1) be repaired without persistent effect, (2) lead to cell death, (3) be fixed into a mutation, or (4) result in chromosomal damage [48]. Therefore, knowledge on the immediate post-exposure levels of DNA damage is vital for predicting the extent of possible subsequent effects in the genome that can have a detrimental outcome. Considering this, our results that show no infliction of significant treatment-related DNA damage in brain tissue are encouraging.

It is easier to interpret the beneficial effect of arbutin thanks to the extensive body of literature on this compound. STE, in contrast, has a complex phytochemical profile. In such a mixture of compounds, each exerts its own specific pattern of action on DNA, while their interactions could also contribute to the final effects observed at the DNA level. Nevertheless, on the basis of only one experiment, it cannot be concluded whether arbutin has stronger genoprotective properties than STE. It would appear that the trends of the obtained values for the comet descriptors were quite similar. This certainly merits further investigation with a larger range of doses.

Here, we will briefly address the strengths and limitations of our research. When referring to the strengths or specific added value of the obtained results, it should be noted that every result concerning a possible beneficial or non-harmful effect of the tested substances at the level of brain tissue is useful for current or future risk assessments.

A specific added value of this study is evidence of non-significantly impaired oxidant/antioxidant status and the level of “primary” DNA damage present in the rat brain cells immediately after termination of treatments with STE or arbutin administered at 200 mg/kg b.w. for 14 vs. 28 days. This is novel information that was not previously known.

Based on the use of elementary toxicological tests, the obtained results could help to guide future research. For instance, as the longer exposure did not produce any alarming toxicological outcomes in rats and considering that the current therapeutic indications of preparations based on *A. uva-ursi* suggest therapeutic use only for short periods of time, by implementing the 3Rs principle, future research on the effects of STE and arbutin on rodent models should not include exposures longer than 14 days.

Our experimental approach also has some limitations. Firstly, the tested doses of STE and arbutin were higher than those that could potentially be achieved through the ingestion of arbutin-rich food or herbal preparations. They were selected for experimental purposes based on the existing literature and recommendations of the European Medicines Agency regarding the use of related herbal preparations based on *A. uva-ursi* [36], though with lower actual doses ingested in the human body. Further, there were several methodological constraints, as we used biomarkers that mostly reflected early detrimental effects of the treatments. Accordingly, the results are valid only within the frame of the proposed model and were not intended to indicate analogous outcomes in humans. Finally, our experimental models (healthy adult male and female rats) could not provide data on the potential neuroprotective effect of the tested substances, which would be very useful to investigate. Similar data currently exist for arbutin tested in vivo at other doses and exposures [6,7,9].

## 4. Conclusions

Based on the results presented here, treatments with arbutin or STE given at daily doses of 200 mg/kg b.w. over 14 vs. 28 days resulted in a high level of biocompatibility with Lewis rat brain tissue. However, these observations are limited to the specific experimental model and cannot be considered relevant for any therapeutic recommendations in humans. In this regard, extensive research at the level of the nervous system should be continued.

The obtained results support the need for further research in order to clarify the safety profile of STE before the development of specific arbutin-rich products potentially useable as nutritional/food supplements. Future research could also be continued in the direction of studying neuroprotective properties using suitable experimental models.

## Figures and Tables

**Table 1:** Experimental schedule.

Experimental Groups (Total N = 48 Rats)	Treatment			
	14-Day Exposure Per Os, Each Day		28-Day Exposure Per Os, Each Day	
Negative control	Double-distilled water		Double-distilled water	
	N = 4 male	N = 4 female	N = 4 male	N = 4 female
Arbutin	200 mg/kg b.w., dissolved in double-distilled water		200 mg/kg b.w., dissolved in double-distilled water	
	N = 4 male	N = 4 female	N = 4 male	N = 4 female
Strawberry tree water leaf extract (STE)	200 mg/kg b.w., dissolved in double-distilled water		200 mg/kg b.w., dissolved in double-distilled water	
	N = 4 male	N = 4 female	N = 4 male	N = 4 female

## Data Availability

All data are available in the manuscript.
